# Health promoting lifestyle of university students in Saudi Arabia: a cross-sectional assessment

**DOI:** 10.1186/s12889-018-5999-z

**Published:** 2018-09-05

**Authors:** Khalid M. Almutairi, Wadi B. Alonazi, Jason M. Vinluan, Turky H. Almigbal, Mohammed Ali Batais, Abdulaziz A Alodhayani, Norah Alsadhan, Regie B. Tumala, Mahaman Moussa, Ahmad E. Aboshaiqah, Razan Ibrahim Alhoqail

**Affiliations:** 1 Department of Community Health Science, College of Applied Medical Sciences, King Saud University, Riyadh, Saudi Arabia; 2 College of Business Administration King Saud University, Riyadh, Saudi Arabia; 3 College of Medicine King Saud University, Riyadh, Saudi Arabia; 4 Department of Family and Community Medicine, College of Medicine, King Saud University, Riyadh, Saudi Arabia; 5 College of Nursing, King Saud University, Riyadh, Saudi Arabia

**Keywords:** Health promoting lifestyle, Students, Healthy behavior, Saudi Arabia

## Abstract

**Background:**

College is a critical time where students are more prone to engage in risky health behaviors known to negatively affect well-being, such as physical inactivity, stress, and poor dietary habits. A health promoting lifestyle is an important determinant of health status and is recognized as a major factor for the maintenance and improvement of health. This study was designed to assess the health-promoting lifestyle of students in health colleges and non-health colleges in Saudi Arabia.

**Methods:**

A total of 1656 students participated in this descriptive cross-sectional study. Data gathering was conducted from November 2016 to February 2017 at King Saud University. Participating students completed a self-reported questionnaire that included questions regarding their demographic characteristics and their health-promoting behaviors.

**Results:**

The majority of participants were females (70.4%), 20% of the participants were overweight and 11.3%, were obese. The analysis showed that there was a significant difference between health colleges and non-health colleges with regards to the factor of health responsibility. Students at both schools were found to have an inadequate level of adherence to recommendations regarding physical activity and healthy eating habits. The analysis also found that majority of the students in both colleges do not attend educational programs on health care. The model shows that gender, type of college, year in school, and family structure were significant predictors of the health lifestyle of students in Saudi Arabia.

**Conclusion:**

The results of the current study indicate that university students are leading unhealthy lives, where the majority of them have unhealthy eating habits and poor physical activity level. Universities are ideal settings for implementing health promotion programs. Therefore, planning and implementing programs to motivate students to be more responsible for their own health, to engage more in physical activity, and to practice healthy eating habits and other forms of wellness are of paramount importance.

## Background

Recently, the increase in the prevalence of non-communicable diseases, such as diabetes, cancer, coronary heart disease, and hypertension has become a global public health concern. It is expected that by 2020, these diseases will be responsible for seven out of every 10 deaths in developing countries [1]. Moreover, an international report has noted that chronic diseases are starting to affect the younger generation instead of only being limited to adults [2]. Obesity in particular is increasing globally at an alarming rate and is estimated to be the fifth leading cause of death worldwide [3]. It is also considered a significant risk factor for other chronic conditions [4]. Sedentary lifestyles and unhealthy eating habits are among the major causes contributing to the obesity epidemic [5]. A generally suggested measure to counter chronic conditions across all age groups is by enhancing health-promoting lifestyles, which would subsequently decrease the rate of disease development [1].

A lifestyle is a way of living that could be considered either healthy or unhealthy depending on personal behavioral choices. According to Walker et al. [6], health-promoting lifestyle has been defined as “a multidimensional pattern of self-initiated actions and perceptions that serve to maintain or enhance the level of wellness, self-actualization and fulfillment of the individual”. Health promoting behaviors include health responsibility, physical activity, nutrition, spiritual growth, interpersonal relations, and stress management [7]. A health promoting lifestyle is an important determinant of health status and is recognized as a major factor for the maintenance and improvement of health [8]. Modifiable health behaviors such as eating habits, physical activity, and smoking are major factors in the development of chronic diseases. Data from both Western and Arab regions has indicated that adolescents and young people are not consuming the recommended servings of fruits and vegetables, are skipping meals, consuming large amount of fats, and not meeting the recommended level of physical activity [9–12]. Furthermore, based from a large national representative study the prevalence of smoking in among Saudis aged 15 years or older 12.2% were current tobacco smokers and 16.0% ever smoked tobacco which males were more likely to smoke than females (21.5% vs 1.1%) [13].

College is a critical time where students are presented with a number of challenges including changes in the social and built environments, developing new social networks, having more behavioral autonomy, and adapting to new schedules [14]. During this period of life, college students are more prone to engage in risky health behaviors known to negatively affect well-being, such as physical inactivity, stress, and poor dietary habits [11, 15]. In Saudi Arabia, a number of studies have revealed negative lifestyles relating to exercise and eating habits among college students [16–19]. Students at Saudi health colleges were also investigated in many studies, it was noted that even this group who are fully aware of the importance of adopting healthy habits, were not adequately adhering to the recommended guidelines [5, 20, 21]. Due to these behavioral factors, Saudi college students are more prone to gain weight, which increases their risk of developing diseases [5, 22]. Healthy behaviors play a major role in wellbeing; exercise has been noted as having potential psychological and physical benefits, and academic achievement has been found to be positively associated with exercise habits [23–25]. Therefore, investigating students’ lifestyles is vital for developing tailored health promotion interventions aimed at improving their quality of life.

This study aims to determine the current status and the determining factors of health promoting behaviors among university students. To our knowledge this is the first study to investigate the differences in these behaviors between students enrolled at health and non-health colleges. Given that students in health majors are exposed to curriculums where they are taught how to maintain their health, we hypothesized that students at health colleges would show a higher level of adherence to healthy lifestyles than those at non-health colleges. Furthermore, this is the first study to investigate the health promoting lifestyle of students applying the Health Promoting Lifestyle Profile (HPLP). Findings of the current study are expected to direct efforts and actions toward enhancing the health of those in need.

## Methods

This descriptive cross-sectional study was conducted from November 2016 to February 2017 at King Saud University (KSU) in Saudi Arabia. KSU is the largest and oldest university in the Kingdom of Saudi Arabia, located in the capital city Riyadh. To conduct this study, ethical approval was provided by the Ethics Committee at the College of Applied Medical Science at KSU (CAMS 003–37/38).

A sample of 1656 students participated in this study; they were enrolled at either the health or non-health colleges of KSU. In this study, health colleges included disciplines such as medicine, nursing, and applied medical sciences, whereas non-health colleges are comprised of the business, computer, and science schools. Convenience sampling was used to recruit students. In addition, the formula by Krejcie & Morgan was used to estimate the sample size of the present study. University professors were asked to distribute a self-administered questionnaire among their students. In order to control for duplicated studies, the survey questionnaire was distributed in each department by one doctor or researcher assigned to that college. Additionally, an online version of the questionnaire was developed via Google Forms and the link was sent to all students enrolled at KSU in this college. The online survey was available for 12 weeks to allow enough time for students to respond. Participation in the study was voluntary and students agreeing to participate were asked to sign a consent form. Moreover, students were assured of the confidentiality and anonymity of the collected data and were informed of their right to both withdraw from the study and skip answering any specific question.

A self-administered questionnaire was used for data collection. It consisted of two sections: the first section was about demographic characteristics including age, gender, type of college, year in school, family structure, residence status, and body height and weight which were transformed into a body mass index score (BMI) (kg/m^2^). BMI scores have been categorized into four groups: underweight (≤18.5); normal weight (18.6–24.9); being overweight (25–29.9); and being obese (≥30) [26].

The second section of the questionnaire included questions from the Health Promotion Lifestyle Profile II (HPLP-II), which consists of a total of 52 items along six subscales [6, 27]. However, in order to encourage the participation of students in this study, to ensure that all questions are being properly answered, and most importantly to ascertain that posed questions are culturally appropriate, the researchers decided to stream it down to three subscales consisting of 26 items. The chosen subscales are the ones known to have the most direct effect on health status, including health responsibility [9 items], physical exercise [8 items] and nutrition [9 items]. The overall score on the scale reflects the level of healthy lifestyle behaviors. All items on the scale were presented positively. Participants responded to each item on a 4-point Likert-type scale (1 = *never*, 2 = *sometimes*, 3 = *often*, and 4 = *always)*. The lowest possible score for the entire scale is 26, and the highest possible score is 104, higher scores on the scale indicated a higher level of health promoting behaviors. Before we started the process of data gathering, the questionnaire was translated into Arabic language by a professional language translator. Also the questionnaire was pilot tested to ensure the reliability, validity, and its appropriateness with respect to cultural relevance here in Saudi Arabia. The Arabic translated HPLP questionnaire had an acceptable Cronbach’s alpha of 0.94. The chosen subscales are the ones known to have the most direct effect on health status, including health responsibility [9 items], physical exercise [8 items] and nutrition [9 items]. The Cronbach’s alpha coefficients of the three subscales that have been used varied between .79 and .87.

Because smoking among female students in Saudi Arabia is a sensitive issue and based from the literature mentioned above the researchers decided to exclude this factor in this study. Moreover, the investigators who were bilingual speakers translated the questionnaire into Arabic and then back translated it into English. The back-translated copy was compared to the original English version and adjustments were made as necessary. The Arabic version was piloted on a sample of students (*N =* 50) to ensure the clarity, understandability and cultural relevance of the items.

### Data analysis

Data analysis was performed using Statistical Package for Social Sciences (SPSS) version 21 (SPSS Inc., Chicago, IL, USA). Descriptive statistics for demographic variables and health –promoting behaviors were reported as frequencies, mean, and standard deviation. The chi-square test was used to determine the difference between health and non-health colleges in each category. Multiple logistic regression was performed to investigate the effect of several predictors on the level of health responsibility. Two tailed significance value was set at *p* < 0.05.

## Results

Table 1 displays findings regarding the participants’ demographics. A total of 1656 students fully completed the surveys. The majority of the participants were female (70.4%) and were 20 years of age or younger (57.4%). Based on their BMI scores, 50% of the students were considered to be of normal weight, and 20.8% having overweight and 11.3% were obese. More than half of the students were enrolled in health colleges and most of the students were either in their preparatory or first year of undergraduate study. Almost all of the participants (76.1%) had a traditional type of family structure consisting of both of their parents, while only 10.4% came from single parent homes. The majority of the participants (94.9%) lived with their families, and only 2.4% lived in university housing. The mean score of health-promoting lifestyle subscales is also presented in Table 1. The highest mean 20.97 ± 4.57 was for an eating habits/nutrition, followed by health-responsibility (18.61 ± 4.56). The mean scores for physical activity behavior dimensions was found to be lower in proportion to the mean score of the other dimensions (16.19 ± 5.12).

**Table 1:** Demographic characteristic of the participants

Variable	Total (*N* = 1656)	
	N	%
Age		
Less than 20	950	57.4
21–30	662	40.0
31 and above	44	2.6
Gender		
Men	491	29.6
Women	1165	70.4
BMI status		
Underweight (Below 18.5)	183	11.1
Normal weight (18.5–24.9)	830	50.1
Overweight (25.0–29.9)	344	20.8
Obese (30 and above)	187	11.3
College		
Health colleges	985	59.4
Non-health colleges	671	40.6
College level		
Prep year & First year	776	46.8
Second year	323	19.3
Third year	292	17.6
Fourth year and fifth year	349	21.1
Family structure		
Parents	1260	76.1
Parents and grand parents	222	13.5
Single father	59	3.5
Single mother	115	6.9
Place of residence		
Family	1572	94.9
Friends	20	1.2
Relatives	15	0.9
University housing	39	2.4
Alone	5	0.3
Diagnosed with health problem		
Yes	212	12.8
No	1439	86.9
Health Promoting Lifestyle profile II (HPLP – II)	Mean ± SD	Minimum – Maximum score
Health Responsibility	18.61 ± 4.56	8–32
Physical Activity	16.19 ± 5.12	6–24
Eating Habits/Nutrition	20.97 ± 4.57	7–28

The differences in health promoting lifestyles among non-health and health colleges were shown in Table 2. The analysis revealed that there was a significant difference across response waves among health colleges and non-health colleges with respect to health responsibility. Only a small proportion of students both in health (11.5%) and non – health colleges (6%) reported any unusual signs or symptoms to a physician or other health professional. Students in health colleges were found to be more proactive in regard to discussing health concerns with health professionals. Nearly 70 % of the students in both colleges never inspect their body for physical changes. Furthermore, the majority of the students in both colleges do not attend educational programs on health care. There was a significant difference in students seeking guidance or counseling between the two college types (*p* = 0.001).

**Table 2:** Difference of health lifestyle among non – health colleges and health colleges

Variable	Non – health colleges (*N* = 671)				Health colleges (*N* = 985)				*P* = value
	Never	Sometimes	Often	Routinely	Never	Sometimes	Often	Routinely	
Health responsibility									
1. Report any unusual signs or symptoms to a physician or other health professional.	97 (14.5)	382 (56.9)	152 (22.7)	40 (6.0)	96 (9.7)	502 (51.0)	275 (27.9)	112 (11.54)	**0.001**
2. Read or watch TV programs about improving health.	216 (32.2)	323 (48.1)	132 (19.7)	0	369 (37.5)	417 (42.3)	183 (18.6)	16 (1.6)	**0.001**
3. Question health professionals in order to understand their instructions	114 (17.0)	209 (31.1)	255 (38.0)	93 (13.9)	141 (14.3)	290 (29.4)	335 (34.0)	219 (22.2)	**0.001**
4. Get a second opinion when I question my health care provider’s advice.	248 (37.0)	285 (42.5)	103 (15.4)	35 (5.2)	303 (30.8)	404 (41.0)	207 (21.0)	71 (7.2)	**0.003**
5. Discuss my health concerns with health professionals.	138 (20.6)	283 (42.2)	184 (27.4)	66 (9.8)	135 (13.7)	386 (39.2)	330 (33.5)	134 (13.6)	**0.001**
6. Ask for information from health professionals about how to take good care of myself.	224 (33.4)	308 (45.9)	104 (15.5)	35 (5.2)	286 (29.0)	440 (44.7)	189 (19.2)	70 (7.1)	**0.048**
7. Inspect my body at least monthly for physical changes/danger signs.	466 (69.4)	161 (24.0)	25 (3.7)	19 (2.8)	621 (63.0)	234 (23.8)	78 (7.9)	52 (5.3)	**0.001**
8. Attend educational programs on personal health care.	383 (57.1)	241 (35.9)	39 (5.8)	8 (1.2)	533 (54.1)	333 (33.8)	92 (9.3)	27 (2.7)	**0.008**
9. Seek guidance or counseling when necessary.	119 (17.7)	280 (41.7)	208 (31.0)	64 (9.5)	130 (13.2)	386 (39.2)	320 (32.5)	149 (15.1)	**0.001**
Physical Activity									
10. Follow a planned exercise program.	225 (33.5)	314 (46.8)	69 (10.3)	63 (9.4)	289 (29.3)	438 (44.5)	136 (13.8)	122 (12.4)	**0.019**
11. Exercise vigorously for 20 or more minutes at least three times a week (such as brisk walking, bicycling, aerobic dancing, using a stair climber).	167 (24.9)	267 (39.8)	125 (18.6)	112 (16.7)	252 (25.6)	360 (36.5)	199 (20.2)	174 (17.7)	0.591
12. Take part in light to moderate physical activity (such as sustained walking 30–40 min 5 or more times a week).	184 (27.4)	276 (41.1)	116 (17.3)	95 (14.2)	289 (29.3)	377 (38.3)	170 (17.3)	149 (15.1)	0.660
13. Take part in leisure-time (recreational) physical activities (such as swimming, dancing, bicycling).	293 (43.7)	261 (38.9)	91 (13.6)	26 (3.9)	366 (37.2)	407 (41.3)	153 (15.5)	59 (6.0)	**0.025**
14. Do stretching exercises at least 3 times per week.	359 (53.5)	211 (31.4)	60 (8.9)	40 (6.0)	483 (49.0)	291 (29.5)	131 (13.3)	80 (8.1)	**0.013**
15. Get exercise during usual daily activities (such as walking during lunch, using stairs instead of elevators, parting car away from destination and walking).	145 (21.6)	249 (37.1)	169 (25.2)	108 (16.1)	213 (21.6)	352 (35.7)	249 (25.3)	171 (17.4)	0.899
16. Check my pulse rate when exercising.	369 (55.0)	6 (0.9)	267 (39.8)	29 (4.3)	484 (49.1)	106 (10.8)	328 (33.3)	67 (6.8)	**0.001**
17. Reach my target heart rate when exercising.	361 (53.8)	200 (29.8)	81 (12.1)	29 (4.3)	476 (48.3)	306 (31.1)	134 (13.6)	69 (7.0)	**0.043**
Eating Habits/Nutrition									
18. Choose a diet low in fat, saturate fat, and cholesterol.	279 (41.6)	262 (39.0)	92 (13.7)	38 (5.7)	361 (36.6)	389 (39.5)	161 (16.3)	74 (7.5)	0.095
19. Limit use of sugars and food containing sugar (sweets).	199 (29.7)	303 (45.2)	124 (18.5)	45 (6.7)	274 (27.8)	430 (43.7)	207 (21.0)	74 (7.5)	0.509
20. Eat 6–11 servings of bread, cereal, rice and pasta each day	171 (25.5)	288 (42.9)	138 (20.6)	74 (11.0)	271 (27.5)	385 (39.1)	209 (21.2)	119 (12.1)	0.525
21. Eat 2–4 servings of fruit each day.	198 (29.5)	365 (54.4)	75 (11.2)	33 (4.9)	299 (30.4)	481 (48.8)	155 (15.7)	50 (5.1)	**0.037**
22. Eat 3–5 servings of vegetables each day.	185 (27.6)	334 (49.8)	103 (15.4)	49 (7.3)	294 (29.8)	447 (45.4)	178 (18.1)	66 (6.7)	0.231
23. Eat 2–3 servings of milk, yogurt or cheese each day.	77 (11.5)	4 (0.6)	436 (65.0)	154 (23.0)	139 (14.1)	143 (14.5)	518 (52.6)	185 (18.8)	**0.001**
24. Eat only 2–3 servings from the meat, poultry, fish, dried beans, eggs, and nuts group each day	99 (14.8)	7 (1.0)	454 (67.7)	111 (16.5)	134 (13.6)	141 (14.4)	552 (56.2)	155 (15.8)	**0.001**
25. Read labels to identify nutrients, fats, sodium content in packaged food.	230 (34.3)	245 (36.5)	107 (15.9)	89 (13.3)	253 (25.7)	341 (34.6)	203 (20.6)	188 (19.1)	**0.001**
26. Eat breakfast.	41 (6.1)	183 (27.3)	150 (22.4)	297 (44.3)	73 (7.4)	277 (28.1)	244 (24.8)	391 (39.7)	0.254

Note: *p*-value significant at *p* < 0.05

With regards to physical activity, a significant difference was found between health and non-health students in following a planned exercise program (*p* = 0.019) and taking part in leisure time and physical activities (*p* = 0.019). Around 35.3% of students in non- health colleges and 37.8% of students in health colleges indicated that they exercise vigorously for 20 min or more at least three times a week. Furthermore, significant differences were found across response waves of performing stretching exercises among the students in non-health and health colleges (*p* = 0.013). However, no significant difference was found between the two groups in regard to practicing light to moderate physical activity.

Analyzing eating habits, significant differences were found between the groups in responses relating to the consumption of fruits, dairies and protein rich foods (*p* = 0.037, 0.001, and 0.001 respectively). Approximately 90% of non-health college students consume 2–3 servings of dairy each day compared to 71.4% of those from health colleges. The majority of the students in both colleges do not choose diets low in fat, and only a small proportion of students in non-health (6.7%) and health colleges (7.5%) limit their intake of sugar. No significant difference was found between the groups in regard to eating breakfast (*p* = 0.254). Approximately 30% and 40% of the students in non-health and health colleges, respectively, reported inspecting and reading food labels to identify different nutrients.

Table 3 presents the factors associated with the healthy lifestyle of students in Saudi Arabia. The socio-demographic variables, including age, gender, college, college level, family structure, and Grade Point Average (GPA) of students were entered and analyzed. The model shows that gender, type of college, year in school, and family structure were significant predictors of the health lifestyle of students in Saudi Arabia. The results show that males were more willing to engage in physical activity than females (*p* = 0.001). The analysis also found that difference in colleges of students was significantly associated with increased likelihood of health responsibility of students. Furthermore, female students had more concern in their diet management and nutrition than male students (*p* = 0.014). There were no significant association in variables of age, place or residence and GPA with on health responsibility, physical activity and diet management or nutrition. Year in school was found to be a significant predictor of physical activity (*p* = 0.001).

**Table 3:** Association of health lifestyle subscales and demographic factors of students in Saudi Arabia

	Health responsibility		Physical activity		Eating habits/Nutrition	
	O.R. (95% CI)	*P*-value	O.R. (95% CI)	*P*-value	O.R. (95% CI)	*P*-value
Age	0.133 (−0.38 to 0.651)	0.615	0.018 (−0.453 to 0.490)	0.940	−0.239 (−0.834 to 0.355)	0.430
Gender	0.518 (−0.105 to 1.141)	0.103	−0.676 (−1.216 to −0.136)	**0.014**	1.307 (0.627to 1.986)	**0.001**
College	−1.391 (−1.902 to − 0.881)	**0.001**	0.988 (0.964 to 1.013)	0.359	0.041 (− 0.512 to 0.594)	0.855
Level	0.041 (−0.175 to 0.257)	0.708	0.345 (0.141 to 0.548)	**0.001**	0.110 (−0.127 to 0.47)	0.363
Family structure	0.044(−0.179to 0.267)	0.699	0.304 (0.034 to 0.504)	**0.028**	0.093 (−0.408 to 0.565)	0.753
Place of residence	0.049 (−0.393 to 0.492)	0.699	0.392 (−0.155 to 0.940)	0.160	0.078 (−0.408 to 0.565)	0.753
Grade Point Average *(*GPA*)*	0.002 (−0.013 to 0.008)	0.655	−0.003 (− 0.015 to 0.010)	0.655	− 0.007 (− 0.018 to 0.003)	0.182

Note: *p*-value significant at *p* < 0.05

## Discussion

Health-promoting lifestyle among adolescents has received an increasing attention worldwide. For example, studies conducted in United States (US) and European countries that evaluated the health-promoting behaviors of university students particularly their physical activity and eating habits or diet [28–30]. However, health-promoting lifestyle among university students in Saudi Arabia are limited. This present study provides a glimpse of health-promoting lifestyle among university students in Saudi Arabia. Our study also assess the differences in these behaviors between students enrolled at health and non-health colleges.

The results showed significant differences between health and non-health colleges with regards to health responsibility. Only a small proportion of students in both colleges reported any unusual signs or symptoms to a physician or other health professional. This result was similar to a finding by Chen et al., 2017 [31]; these results could be explained by the fact that university students are relatively young and may not notice any unusual signs and symptoms, worry about their health status, or consider themselves at risk. Another interesting finding of this study was most of the students in both colleges had rarely asked questions to health professionals in order to understand their instructions or even discussed health concerns with them. Acknowledging the role of health professionals in providing health information and education as well as discussing health concerns illustrated the students’ health responsibility in adopting a healthy lifestyle [32]. Considering the importance of educational programs and support activities, our results showed that majority of the student in both colleges did not attend educational programs on health care which was contrary to a previous study in which university students participated in educational programs and some sort of support activities on health care [33].

Another significant difference between students in non-health and health colleges was noted in seeking guidance or counseling. Majority of students in health colleges reported seeking professional counseling or guidance when needed. These personal health attributes were previously noted as definitive indicators of the health status of students [34]. Therefore, health promotion programs should place an emphasis on educating students in non-health colleges about the importance of counseling in maintaining their health.

In relation to the level of physical activity among students, our findings revealed a significant difference between colleges; more students in health colleges followed a planned exercise program and took part in leisure time and physical activities than in non-health colleges. This might be due to the fact that these students have better health knowledge, which was reflected positively in their exercise behavior. Our results indicated that a significant proportion of students exercised vigorously for 20 or more minutes at least three times a week. This result was contrary to previous studies, which reported insufficient levels of vigorous recreational physical activity and indicated increased suboptimal health status among participants [35–38]. The findings suggested that frequent engagement with recreational activities may be associated with better reports of health, physical and psychological wellbeing. In similar studies, those who exercised regularly showed better physical fitness, and perceived physical and psychological health [39]. Furthermore, it was revealed that there were significant differences across response waves of doing stretching exercise at least 3 times per week among the students in non-health and health colleges. This result is similar to a previous study where nursing students were tested for their body flexibility but not limited to stretching exercise [39]. Meanwhile, no significant difference was found between students of both colleges and their response towards exercising during usual activities. Moderate-intensity and vigorous-intensity exercises are required to achieve cardiovascular fitness among university staff and students, and those who are physically active are more likely to pay attention to their health [40]. In our study, students in both colleges did not take part in light to moderate physical activity. This result was similar to a study in which female nursing students in Korea denied participating in regular exercise [41].

In terms of health-promoting lifestyle subscale eating habits, our results showed a significant difference in responses between students in non-health and health colleges in consumption of nutritious foods each day. The majority of the students in both colleges did not choose diets low in fat, saturated fat and cholesterol have shown that the majority of students are living with their families and such a setting has been noted to be promoting of good dietary choices [42]. Despite this, the vast majority of students regardless of their major did not opt for diets that are low in fat, which might indicate that they are consuming fast food products at higher rates. This result is consistent with other local studies were students were found to have high intake of high fat food [5, 20]. This might be explained by the strong influence exerted by external factors on dietary choices, such factors include but are not limited to peers and the university environment where students spend a great deal of time.

All students in our study reported low intake of fruits and vegetables; these results are in line with earlier findings reported in local and international studies [16, 43, 44]. In contrast, research on Chinese students has showed higher consumption of fruits and vegetables [45]. This might be explained by the traditional eating habits in this region where fruits and vegetables are main components in their dishes; on the other hand, the traditional Saudi diet is known to lack these essential ingredients. Additionally, previous research has noted lack of time, lack of knowledge and limited access to nutritious foods as barriers to healthy eating among university students [46]. Medical students as well as those enrolled at health colleges are known to have time management issues due to their tight study schedules; however, in our study even students at non-health colleges were not eating the recommended amount of fruits and vegetables. Further investigation is needed to ascertain the potential barriers confronted by our sample and consequently address them effectively.

Our present study revealed that the vast majority of students are eating breakfast, which was consistent with the result of a similar study on Saudi students [5]. Also, the results showed a significant difference between colleges in relation to reading food labels. Students at health colleges reported more adherences to this practice; this could be due to the fact that these students are exposed through their studies to valuable nutritional knowledge in this regard. This finding indicates that more guidance and educational interventions should be provided to students enrolled at colleges where they are less likely to be informed about food contents.

These results emerged at this period during the university stage of students as risky health and nutrition activities such as unhealthy diet practices [41, 47]. More often, this health risk is primarily the result of transitioning from, and secondary to tertiary education secondary to increase poor dietary choices [48, 49]. These unhealthy dietary deeds among participants raised a noteworthy health concern for being at risk for malnutrition and obesity. It was an unexpected result that even students in health colleges reported unhealthy diet practice. The problem is that in spite of their knowledge about healthy eating, students still will choose diets that are high in sugars and fats. This finding indicates that more needs to be done to understand what compels students to make lifestyle choices that go against their better judgment. More research into how the university environment impacts the decision making of students may be helpful in closing that gap.

In the present study, the model showed that gender, college, year in school, and family structure were significant predictors of a healthful lifestyle of university students in Saudi Arabia. The results also showed that males are more willing to engage in physical activity than females. This is similar to other studies in Japan and Portugal where male participants reported higher physical activity than their female counterparts [50, 51]. This can be explained by cultural considerations such as the fact that Saudi females have more restrictions placed on them when it comes to doing physical activities than Saudi males. The analysis also found that differences in colleges were significantly associated with increased likelihood of health responsibility of students. Furthermore, female students were more concerned about diet and nutrition than male students. The findings were similar to previous studies indicating that females were more aware of health related concepts and acknowledged the connection between nutrition and health much more than males [45, 50]. Year in school was found to be a significant predictor for physical activity and exercise. Similar to a previous study in Japan, general education courses like physical education are offered in the preparatory year in Saudi Arabia and are no longer included in the higher college levels [50]. This may be one of the reasons contributing to the increasingly sedentary lifestyle of university students in Saudi Arabia. With regard to family structure, it was found to be a significant predictor of participants’ physical activity level. This result was similar to previous studies signifying that a favorable health action is shown when both parents have higher educational level and their children seek their support [33, 52].

Several limitations should be addressed in future researchers related to this study. The cross-sectional design of this current study did not explain causation and changes over time in health lifestyles among the participants which posed one limitation. Another limitation of the study was the sample was heavily weighted towards towards female students, this can be explained that the majority of the students who participated were females and have more concern about the study. In addition, as all information gathered in this study was based on self-reporting, it is possible that the university students gave answers they thought the researchers wanted to hear. Furthermore, the results cannot be generalized to university students across the kingdom because its sampling technique is limited to one university.

## Conclusion

The results of the current study indicate that university students are leading unhealthy lives, where the majority of them have unhealthy eating habits and poor physical activity level. A sense of health responsibility needs to be further developed among students in non-health colleges; such personal attribute plays an essential role in early diagnosis of health problems. Health promotion interventions targeted at university students are of paramount importance, as these could help in establishing healthy habits that will be adopted for life. Our findings highlight the need to further investigate the barriers and potential facilitators for a healthy lifestyle at the university environment. Moreover, it is essential to design curriculums and counseling services aimed at providing students with the knowledge, support and empowerment needed to make informed choices pertaining to their health. Finally, health behaviors are known to be of complex nature, thus, coordinated efforts directed at all levels of influence including the individual, environment, community, and policy levels are necessary to create contexts where healthy lifestyles thrive and become habits.

The current study explored the health lifestyles of university students regarding their health responsibility, physical activity, and nutrition. As universities are ideal settings for implementing health promotion programs, planning and implementing those programs to motivate students to be more responsible for their own health, engage in regular physical activity, and practice healthy diet with the purpose of promoting health and preventing diseases are of paramount importance. Thus, it can be concluded that developing and implementing goal-oriented programs to promote health responsibility, physical activity and nutrition may promote healthy lifestyles among university students. Applications like ‘Healthy University’ and ‘Universities Humanizing Health’ are also suggested to enhance health lifestyle awareness of students.

## Abbreviations

BMI: Body Mass Index
CAMS: College of Applied Medical Sciences
GPA: Grade point average
HPLP-II: Health Promotion Lifestyle Profile – II
KSU: King Saud University
SPSS: Statistical Package for Social Sciences
US: United States
